# Impact of α-Synuclein Fibrillar Strains and β-Amyloid Assemblies on Mouse Cortical Neurons Endo-Lysosomal Logistics

**DOI:** 10.1523/ENEURO.0227-21.2022

**Published:** 2022-05-11

**Authors:** Qiao-Ling Chou, Ania Alik, François Marquier, Ronald Melki, François Treussart, Michel Simonneau

**Affiliations:** 1 Université Paris-Saclay, École Normale Supérieure Paris-Saclay, Centre National de la Recherche Scientifique, CentraleSupélec, LuMIn, Gif-sur-Yvette 91190, France; 2 Laboratory of Neurodegenerative Diseases, Institut François Jacob (MIRCen), Centre National de la Recherche Scientifique, Commissariat à l’Énergie Atomique et aux Énergies Alternatives, Université Paris-Saclay, Fontenay-aux-Roses Cedex 92265, France; 3 Département d’Enseignement et de Recherche en Biologie, École Normale Supérieure Paris-Saclay, Gif-sur-Yvette 91190, France

**Keywords:** α-synuclein, β-amyloid assemblies, endosome, intraneuronal transport, lysosome, mouse cortical neuron

## Abstract

Endosomal transport and positioning cooperate in the establishment of neuronal compartment architecture, dynamics, and function, contributing to neuronal intracellular logistics. Furthermore, dysfunction of endo-lysosomal has been identified as a common mechanism in neurodegenerative diseases. Here, we analyzed endo-lysosomal transport when α-synuclein (α-syn) fibrillar polymorphs, β-amyloid (Aβ) fibrils, and oligomers were externally applied on primary cultures of mouse cortical neurons. To measure this transport, we used a simple readout based on the spontaneous endocytosis in cultured neurons of fluorescent nanodiamonds (FNDs), a perfectly stable nano-emitter, and the subsequent automatic extraction and quantification of their directed motions at high-throughput. α-Syn fibrillar polymorphs, Aβ fibrils, and oligomers induce a 2-fold decrease of the fraction of nanodiamonds transported along microtubules, while only slightly reducing their interaction with cortical neurons. This important decrease in moving endosomes is expected to have a huge impact on neuronal homeostasis. We next assessed lysosomes dynamics, using LysoTracker. Neurons exposure to Aβ oligomers led to an increase in the number of lysosomes, a decrease in the fraction of moving lysosome and an increase in their size, reminiscent of that found in APP transgenic model of Alzheimer’s disease. We then analyzed the effect of α-syn fibrillar polymorphs, Aβ fibrils, and oligomers on endosomal and lysosomal transport and quantified directed transport of those assemblies within cortical neurons. We report different impacts on endosomal and lysosomal transport parameters and differences in the trajectory lengths of cargoes loaded with pathogenic protein assemblies. Our results suggest that intraneuronal pathogenic protein aggregates internalization and transport may represent a target for novel neuroprotective therapeutic strategies.

## Visual Abstract

**Figure:**
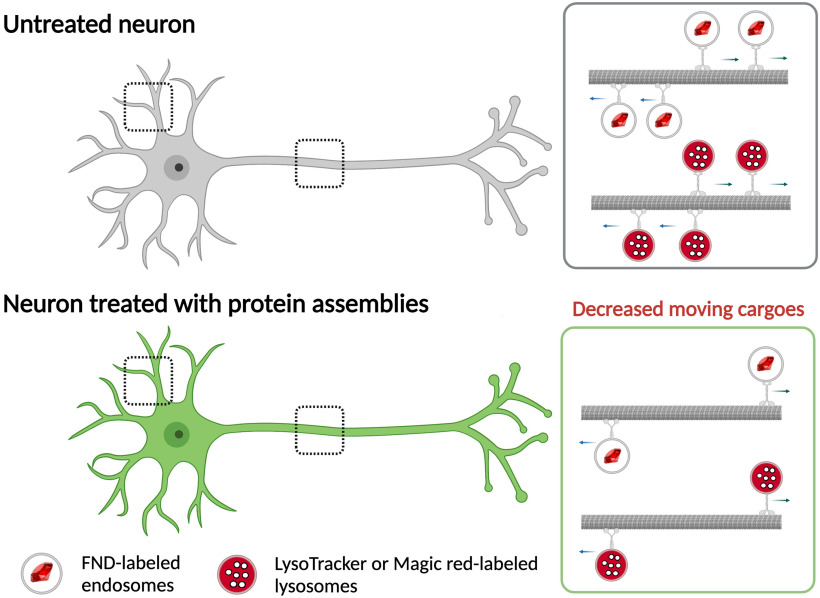

## Significance Statement

Neurodegenerative diseases are characterized by the deposition of protein aggregates. These proteins exert a broad range of neuronal toxicity. Defects in endo-lysosomal traffic are increasingly viewed as key pathologic features of neurodegenerative diseases, likely contributing to synaptic dysfunction and ultimately neuronal death. Here, we measured by fast fluorescence video-microscopy the endosomal and lysosomal dynamics in the branches of primary culture of mouse cortical neurons after externally applying α-synuclein (α-syn) fibrillar polymorphs (fibrils or ribbons) and β-amyloid (Aβ) assemblies (oligomers or fibrils). We provide significant insight into the differential effects of these pathogenic protein assemblies on endosomal and lysosomal transport, and also reveal distinct transport characteristics of the compartments loaded with these protein assemblies compared with endosome ones.

## Introduction

Impairment of axonal transport has recently emerged as a factor shared by several neurodegenerative disorders (Morfini et al., 2009; Millecamps and Julien, 2013). Early impact on intraneuronal transport has been thus proposed as a phenotypic trait common to neurodegenerative diseases such as Alzheimer’s, Huntington’s, and Parkinson’s diseases (Stokin et al., 2005; Volpicelli-Daley et al., 2014; Saudou and Humbert, 2016). There is compelling evidence that abnormal protein accumulation in the brain is a key pathophysiological mechanism underlying the neurotoxicity observed in these age-related disorders (Golde et al., 2013; Soto and Pritzkow, 2018). Selective aggregation of misfolded proteins is a hallmark of these neurodegenerative diseases (Saez-Atienzar and Masliah, 2020). An important level of complexity comes from the fact that different species of the same molecules, such as oligomers and fibrils, contribute to a whole spectrum of toxicities (Alam et al., 2019).

Few studies have compared, within the same neurons, fibrillary and oligomeric α-synuclein (α-syn) and β-amyloid (Aβ) traffic, which is known to be involved in Parkinson’s and Alzheimer’s diseases, respectively. Brahic et al., 2016; demonstrated for instance that α-syn, Aβ_42_, and HTTExon1 fibrils are transported anterogradely and retrogradely with different efficiencies in axons of mouse primary neurons grown in microfluidic chambers. Here, we thoroughly quantified the impact of two α-syn fibrillar polymorphs namely fibrils (α-synF) and ribbons (α-synR), Aβ_42_ fibrils (AβF) and oligomers (AβO) on endosomal and lysosomal transports in primary cultures of mouse neurons. To measure this transport and investigate finely its parameters, we relied on our previously established method (Haziza et al., 2017), in which we let perfectly stable and nontoxic fluorescent nanodiamonds (FNDs) being spontaneously internalized by neurons in endosomes, then follow their displacement by fast video-microscopy and finally apply to the videos our analysis pipeline to extract and analyze single particle trajectories automatically. Using fluorescently-labeled α-syn and Aβ assemblies, we conducted the same investigations on their own intraneuronal transport.

Our data allow to address three complementary questions: (1) do α-synF, α-synR, AβF, and AβO influence the fraction of cargoes moving along the microtubules; (2) do they impact the dynamics of intracellular endosomal and lysosomal transport?

We show here that all pathogenic proteins assemblies reduce the fraction of endosomes moving along microtubules and impact some of their transport parameters. Furthermore, lysosomes properties (number, fraction of lysosomes moving and transport parameters) are also affected by AβO. Finally, our data indicate that cargoes loaded with α-synF, α-synR, AβF, or AβO are transported differently from endosomes, considered as control cargoes, which suggests distinct molecular characteristics of cargo-motor assemblies.

## Materials and Methods

### Production of α-syn fibrillar assemblies, Aβ fibrils, and oligomers

The expression and purification of human wild-type (WT) α-syn was performed as previously described (Ghee et al., 2005). Pure WT α-syn was incubated in buffer A to obtain the fibrillar polymorph “fibrils” α-synF (50 mm Tris-HCl at pH 7.5, 150 mm KCl) and in buffer B for “ribbons” α-synR (5 mm Tris-HCl at pH 7.5) at 37°C under continuous shaking in an Eppendorf Thermomixer set at 600 rotations per minute (rpm) for 4–7 d (Bousset et al., 2013). The fibrillar α-syn polymorphs were centrifuged twice at 15,000 × *g* for 10 min and resuspended twice in PBS at 3 g/l (or 215 μM) before labeling with ATTO 488 NHS-ester (#AD 488–3, Atto-Tec) fluorophore following the manufacturer’s instructions using a protein:dye ratio of 1:2. The labeling reactions were arrested by addition of 1 mm Tris (pH 7.5). The unreacted fluorophore was removed by a final cycle of two centrifugations at 15,000 × *g* for 10 min and resuspensions of the pellets in PBS. This labeling protocol typically yields ≥1 ATTO molecule incorporated per α-syn monomer on average as previously demonstrated (Shrivastava et al., 2015). The assemblies were examined by transmission electron microscopy after adsorption on 200 mesh carbon-coated electron microscopy grids and negative stained with 1% uranyl acetate before and after fragmentation using a JEOL 1400 electron microscope (JEOL).

The expression and purification of Met-Aβ 1–42 was performed as described (Walsh et al., 2009). Aβ was assembled in PBS, at 4°C or 37°C without shaking for 2 or 24 h to obtain oligomers AβO or fibrils AβF, respectively. The two kinds of assemblies were labeled with ATTO 488 NHS-ester at a protein:dye ratio of 1:2. The labeling reactions were arrested by addition of 1 mm Tris at pH 7.5. For fibrillar Aβ, the unreacted fluorophore was removed by two cycles of centrifugation and resuspension of the pelleted fibrils in PBS as described for α-syn. For oligomeric Aβ, the oligomers were separated from the monomeric and fibrillar forms of the protein by size exclusion chromatography on a Superose 6 HR10/300 column (GE Healthcare, Life Sciences) equilibrated in PBS pH 7.4 at a flow rate of 0.5 ml/min. Elution was monitored by measuring absorbance at 280-nm wavelength. The Superose 6 column was calibrated with Dextran blue (over 2200 kDa), thyroglobulin (670 kDa), β-amylase (200 kDa), BSA (66 kDa), and carbonic anhydrase (29 kDa) standards (Sigma-Aldrich).

### Primary mouse cortical neuron cultures

We used commercial primary mouse cortical neurons (ref. Invitrogen A15586, ThermoFisher) because the provider quality check guarantees a purity of 98% of neurons. The cells were grown on high optical quality glass coverslips (high-precision 170 ± 5 μm thick, 18-mm diameter, ref. 0117580, Marienfeld GmbH). The coverslips are first cleaned with 70% ethanol, rinsed with water for injection (ref. A128730, ThermoFisher) and exposed during 1 h to UV light. They were then coated with 0.1 mg/ml poly-L-ornithine (ref. P3655, Sigma-Aldrich Merck KGaA) and placed for 2 h in an incubator set at 37°C, then rinsed twice with water and let dry at biological hood for 1 h. We plated an amount of 6 × 10^5^ primary mouse cortical neurons on each coated coverslip, which was then put at the bottom of a 6-wells plate, each well being finally filled with 3 ml of neurobasal phenol red-free medium (ref. 12348017, ThermoFisher) containing 0.5 mm GlutaMax (ref. 35050061, ThermoFisher), 2% B-27 (ref. 17504044, ThermoFisher) and 1% PenStrep (ref. 15070063, ThermoFisher). The 6-well plate was then placed in an incubator at 37°C and 5% CO_2_. Half of the volume of the medium was replaced with fresh medium 24 h after plating. We made the subsequent medium changes every 3 d to reduce glutamate toxicity. Neurons were grown until 21 d in culture (DIC).

### Exposure of mouse cortical neurons to α-syn fibrillar assemblies, Aβ fibrils, or oligomers

In all the measurements dealing with (1) the impact of pathogenic protein assemblies on endosomal-lysosomal transport; (2) their colocalization with FND-labeled compartment or lysosomes; or (3) the tracking of their intraneuronal transport by fluorescence video-microscopy, cortical neurons were incubated with either 0.2 μm ATTO 488-labeled α-synF or R, or 1 μm ATTO 488-labeled AβF or AβO.

α-SynF or R were added at 24, 48, or 72 h before observations, while the addition time was either 24 or 48 h for ATTO 488-labeled AβF or AβO. The video acquisitions of all the experiments were performed at 21 DIC.

### Washing protocol to test the protein assemblies interaction with the neuron membrane

The coverslips with the culture attached to them were extracted from the well and flushed with PBS first and then twice with culture medium, before FND internalization was conducted, following the procedure described in the next paragraph.

### Intraneuronal transport cargo labeling

To evaluate the endosomal transport parameters, we relied on our FND assay (Haziza et al., 2017). We used commercially available sized 35 nm FND (brFND-35, FND Biotech). Each NP contains an average of 15 nitrogen-vacancy emitters displaying a peak emission wavelength around 700 nm and a full-width at half-maximum of ≈100 nm. This far-red emission allows also to investigate the colocalization of green-emitting ATTO 488-labeled neurodegenerative-disease related species with FND-labeled cargoes. FND were internalized in cortical neurons just before the transport analysis, at DIC21. Each culture coverslip was removed from the six-well plate containing maintaining medium and put in contact with 400 μl of fresh culture medium to which we added 2 μl of stock solution of FNDs (1 mg/ml), reaching a final FND concentration of 5 μg/ml. After 10-min incubation, the extra FND-containing medium was absorbed by a wiper sheet and the coverslip was placed back to the dish containing the old maintaining medium. The culture was then placed back during 20 min in the incubator before the video acquisition.

To measure lysosomal transport or investigate the colocalization of neurodegenerative disease-related species with lysosomes, cortical neurons were stained at DIC21, just before the observation, with LysoTracker Deep Red (ref. L12492, ThermoFisher) or Magic Red Cathepsin B substrate (ref. ICT937, Bio-Rad). These dye molecules have an emission spectrum within the similar range than the one of FND. The coverslip was removed from maintaining medium and incubated with prewarmed (37°C) culture medium containing 50 nm LysoTracker or Magic Red (1:20 dilution) for 1 h. The probe-containing medium was replaced with the old maintaining medium and followed by video acquisition.

### Pseudo-total internal reflection (TIRF) live-cell video-microscopy

Pseudo-TIRF illumination was implemented on an inverted microscope (Eclipse Ti-E, Nikon) as described in details in (Haziza et al., 2017). The whole microscope is enclosed in a cage incubator (Okolab) to maintain temperature at 37°C. For the intraneuronal transport recording, each coverslip supporting the neuron culture is mounted at the bottom of a Ludin chamber (type 1, Life Imaging Service), installed inside the environmental chamber (in which 5% partial CO_2_ pressure and 100% hydrometry is maintained) having a hole at its bottom allowing direct optical access of the microscope objective to the coverslip. We used a ×100 magnification and 1.49 numerical aperture immersion oil objective (CFI Apo TIRF ×100 Oil, Nikon), compatible with differential inference contrast mode. Field of views (FoVs) of interest of the neuron cultures were selected in white-light illumination differential inference contrast mode. Two continuous-wave lasers are coupled to the microscope and fluorescence was recorded on a cooled EMCCD array detector (DU-885K-CS0, Andor Technologies) of 1004 × 1002 pixels, with 80-nm pixel size in the sample plane. Two-minute duration videos were acquired at 20 full frame/s rate, large enough to be able to detect short pausing duration in cargoes displacements. EMCCD parameters were selected to provide the largest signal-to-background ratio for FND label tracking at the selected frame rate, leading to EM gain of 90, preamplification gain 3.8×, and digitalization speed of 35 MHz. FNDs and LysoTracker fluorophore were excited with a diode-pumped solid-state laser at a wavelength of 561 nm (SLIM-561-100, Oxxius S.A.), while ATTO 488 dye was excited with a laser diode emitting at a wavelength of 488 nm (LBX-488–200-CSB-PP, Oxxius S.A.). Each excitation laser power was adjusted so that the detection dynamic range of all channels was identical for the above mentioned fixed EMCCD settings. This leads to 561-nm laser excitation power of 60 mW for FND, 200 μW for LysoTracker and 1 mW for Magic Red, and to 488-nm laser excitation power of 200 μW for ATTO 488 dye.

To perform two-color acquisitions and record simultaneously FND (or LysoTracker) and ATTO 488 we combined the two laser beams with a dual-band dichroic filter (ref. Di01-R488/561, Semrock), and placed a dual imaging system (W-VIEW GEMINI, Hamamatsu) in front of the EMCCD array detector. This system splits half of the detection FoV in two color channels with a dichroic beamsplitter (FF560-FDi01, Semrock) and projects each color on half of the array detector, further preceded by bandpass detection filters (red channel: HC697/75, Semrock; green channel: ET525/50, Chroma Corporation). The result is that each frame of the video contains the same rectangular FoV (1004 × 501 pixels) in green (ATTO 488) and red (FND and LysoTracker) emission range, allowing to identify spots that colocalize dynamically.

### Video processing and intraneuronal transport quantification

Two programs written in python were developed to extract quantitative parameters from videos automatically. The first one relies on Trackpy 0.4.2 package (Allan et al., 2019), from which it uses two functions: “locate” to identify isolated spots in each fluorescence frame and fit them with gaussians, and “link” which connects the spots between frames to form trajectories using Crocker–Grier algorithm (Crocker and Grier, 1996). Transport parameters are then calculated with a second program which first parses each trajectory into “go” and “stop” phases based on the confinement ratio calculation as described in (Haziza et al., 2017), with a threshold set to 0.8 for this parameter, the “go” phase phases corresponding to confinement ratio larger to this value. Four main transport parameters are extracted for each trajectory: velocity, which is the average speed of all go phases; run length: average distance traveled during all go phases; pausing time: average duration of the stop phases, and pausing frequency (events/min). In addition to these four main parameters, we also calculated the total length of the trajectory as the sum of all run lengths during go phases.

### Lysosomes size estimation

To get an estimate of the lysosome size from the diffraction limited fluorescence images, we considered the LysoTracker spots as the result of the convolution of the microscope point spread function (assimilated to a Gaussian of standard deviation, SD, $\sigma_{PSF}$) and the lysosome assimilated to a symmetrical Gaussian of SD $\sigma_{L}$. The result of this convolution is also a Gaussian of SD $\sigma_{T}$, related to $\sigma_{L}$ and $\sigma_{PSF}$ by $\sigma_{T}{}^{2}=\sigma_{PSF}{}^{2}+\sigma_{L}^{2}$. $\sigma_{T}$ is the so-called “size” output of the Trackpy locate function. The knowledge of $\sigma_{T}$ and $\sigma_{PSF}$ allows the derivation of $\sigma_{L}$. We then defined the lysosome “diameter” $d_{L}$ as the full width at half maximum of its Gaussian approximation, inferred by $d_{L}=2\sqrt{2\ln\;2}\sigma_{L}$. For the PSF size $\sigma_{PSF}$ we took the measured value of the smallest FND spot observed in several trajectories with our microscope. This value was $\sigma_{PSF}$=112 nm, consistent with the theoretical Airy radius $\rho_{A}$=286 nm (diffraction limit at 700-nm maximum emission wavelength for the 1.49 numerical aperture objective used), and the empirical relation $\sigma_{PSF}=\frac{\rho_{A}}{3}$, giving here an experimental PSF SD of 286/3 = 95 nm.

### Quantification of fluorescence intensity of ATTO 488-labeled α-synF in cortical neuron branches

We quantify the fluorescence intensity from the first frame of the green channel videos, using Fiji ImageJ software (Schindelin et al., 2012). We first identify from the differential inference contrast image some well-separated and mainly straight neuronal branches, that we surround with the region-of-interest (ROI) polygonal selection tool as close as possible to the branch to include all the fluorescence signal, over a length of 30 μm. We then used the Analyze function to measure the average intensity counts per pixel in the defined ROI, to which we subtract the average background counts, measured after having moved the ROI in a region without branches.

### Data representation and statistical analysis

All bar plots display the ±SEM of the distribution. Box plots display the median value as the horizontal line within the box whose limits are 25% and 75% percentiles; bottom and top horizontal lines correspond to 10^th^ and 90^th^ percentiles. As all the data compared between two conditions were random and normally distributed but with unequal variance (as tested with a *F* test), we performed the relevant comparison test which is the nonparametric Wilcoxon Mann–Whitney two-tailed (implemented in Igor Pro 8, Wavemetrics Inc.). Stars referred to the following *p*-value significance level: *p <* *0.05, **p <* *0.01, ***p <* *0.001.

## Results

### Quantification of intraneuronal transport using FNDs

**Figure 1.**
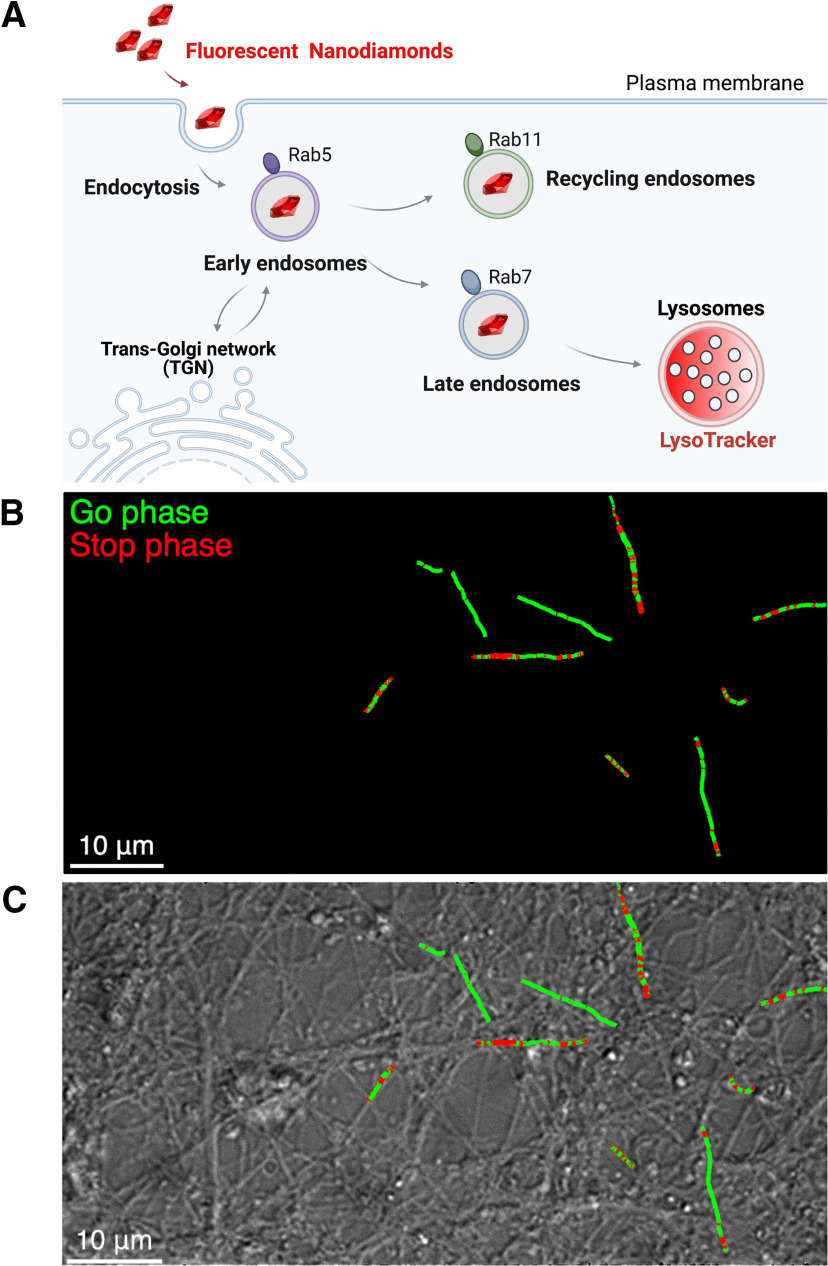
Recording FND trajectories in mouse cortical neurons at DIC21. ***A***, Schematic representation of the cargoes that are tracked thanks to FND, at different stages after their endocytosis. We previously showed (Haziza et al., 2017) that FND are present in cargoes at different stage of their lifetime after endocytosis, as shown by colocalization measurements with specific membrane protein markers: Rab5 for early endosome; Rab7 for late endosome; Rab11 for recycling endosome; LysoTracker for the lysosome. ***B***, Illustration of FND trajectories with go (green) and stop (red) phases. ***C***, Differential interference contrast images of cortical neurons overlapped with 10 representative trajectories. Scale bar: 10 μm.

We quantified the intraneuronal transport with our FND tracking assay (Haziza et al., 2017). We first used a simple readout consisting in counting the number of FND detected in FoVs of size 40 × 80 μm during 2 min. We selected each FoV based on the criteria that it contains approximately the same density of neuronal branches, as estimated from differential interference contrast images (Fig. 1). Our incubation protocol was designed to strongly limit any nonspecific interactions of FND, like their attachment to the coverslip supporting the culture, and favor their interaction with neuron membranes and their subsequent internalization in endosomes (Haziza et al., 2017). The perfectly stable fluorescence of FND allows to reconstruct the endosome trajectories accurately and identify “go” and “stop” (none or very slow motion) phases (Fig. 1*B*) in the transport of FND-labeled endosomes along neuronal branches as observed in differential interference contrast microscopy (Fig. 1*C*).

### α-SynF and R affect the number of cargoes transported along microtubules without major changes in trajectory length

Primary cultures of mouse cortical neurons were incubated at DIC20 with α-synF or R at a concentration of 0.2 μm for 24 h. The intraneuronal transport in these cultures was investigated at DIC21.

**Figure 2.**
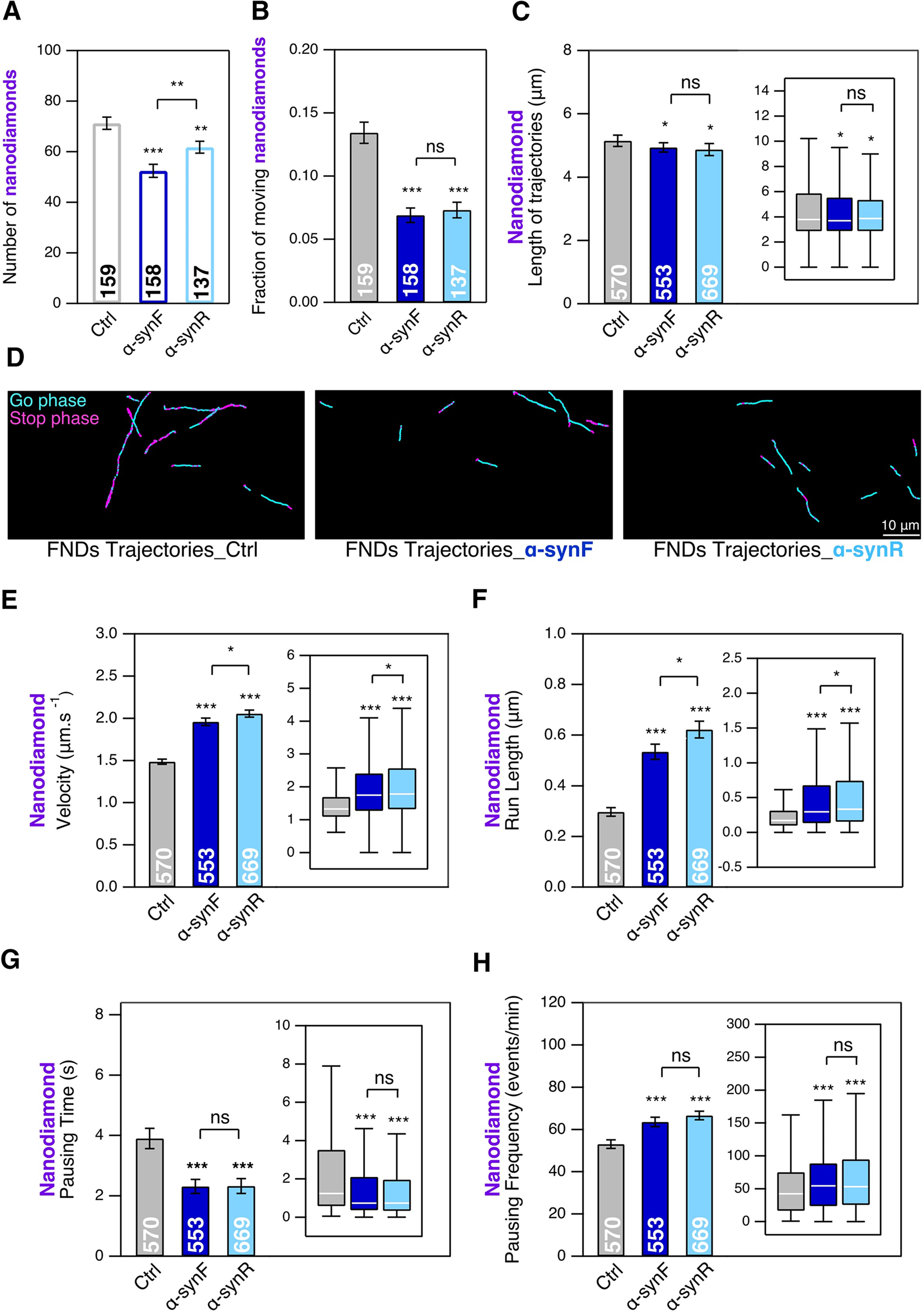
Effect of α-synF or R on the mobility of endosomes and their transport as measured by tracking FND-containing cargoes in mouse cortical neurons at DIC21; 24-h exposure to α-synF or R at 0.2 μm concentration, compared with nothing added control (Ctrl). ***A***, Number of FNDs detected per FoV of 40 × 80 μm size during 2 min of observation. ***B***, Fraction of FNDs-containing cargoes having a directed motion. ***C***, Length of FND trajectories. ***D***, Examples of FND trajectories. Scale bar: 10 μm. ***E*–*H***, Comparison of four transport parameters: (***E***) curvilinear velocity, (***F***) run length, (***G***) pausing time, and (***H***) pausing frequency. The number within each bar represents the total number of FoVs (***A***, ***B***) or trajectories (***C***, ***E*–*H***) analyzed from *n *=* *8 coverslips (4 independent cultures). Insets, Box-plots representation of the same dataset. * and *** mean *p*<0.05 and *p*<0.001 respectively; ns: non significant. See also Extended Data Figures 2-1, 2-2.

Both exposures to α-synF or R led to a small decrease (26% for α-synF and 13% for α-synR) in the number of FND (moving or not) present in each FoV, indicating that both fibrillar polymorphs impact the FND binding to neuronal membrane and their transport dynamics within neurons (Fig. 2*A*). Indeed, if we consider the fraction of these FND having a directed motion, corresponding to those being first internalized in endosomes and then taken in charge by molecular motors, we observed that it decreases by 49%, on exposure of neurons to α-synF and 45% in the case of α-synR (Fig. 2*B*). The unknown mechanism involved in such a large decrease in the number of cargoes transported along microtubules is expected to impair the functions of cortical neurons.

10.1523/ENEURO.0227-21.2022.f2-1 Extended Data Figure 2-1 FNDs interaction with neurons and fluorescence intensity of ATTO 488-labeled α-synF with and without (w/o) washing; 24-h exposure of neurons to α-synF at concentration of 0.2 μm, compared to nothing added control. A, Number of FNDs detected per FoV of 40 × 80 μm during 2 min of observation for the different conditions. Ctrl: no addition of α-synF; α-synF Wash: addition of α-synF during 24 h and washing of the culture just before addition of FND tracers. Inset, Box-plots representation of the same dataset. B, Fraction of FNDs-containing cargoes having a directed motion. The numbers inside the bar in A, B represent the total number of FoVs analyzed from n = 2 coverslips (from one culture). C, Example of FND trajectories in the different conditions. Scale bar: 10 μm. D, Average fluorescence intensity of ATTO 488-labeled α-synF evaluated for 30-μm-long branches, with or w/o washing (n = 25 branches from 25 FoVs). E, Examples of first frames of TIRF video-microscopy of ATTO 488-labeled α-synF decoration of DIC21 cortical neurons with (top) or w/o (bottom) washing. Scale bar: 10 μm. Download Figure 2-1, TIF file.

10.1523/ENEURO.0227-21.2022.f2-2 Extended Data Figure 2-2 Summary of the effect of different protein assemblies on the transport parameters of endosomes (A), LysoTracker-labeled lysosomes (B), and Magic Red-labeled lysosomes (C). Download Figure 2-2, TIF file.

In order to determine whether this important reduction of FND moving fraction is related to the binding of the protein assemblies to the neuronal membrane, possibly leading to a reduced endocytosis, we tried in the case of α-synF to wash away the aggregates just before the addition of FND (Materials and Methods), but we found that the interaction of the nanodiamonds with neuron did not change in washed-neuron condition compared with unwashed (Extended Data Fig. 2-1*A–C*); in particular, we did not observe differences in the fraction of moving FND. We concomitantly measured the amount of ATTO 488-labeled assemblies along the neuronal branches (as quantified by the dye fluorescence intensity) and could not see any differences before and after washing (Extended Data Fig. 2-1*D*,*E*), which is in agreement with FND-neuron interaction results, and indicate a strong binding of α-synF to the neuronal membrane.

Using our established FND-based intraneuronal transport assay (Haziza et al., 2017) we detected and quantitatively analyzed the alternation of movement and pause phases of intraneuronal cargoes motion. We first measured the length of trajectories (see Materials and Methods) for control FND and FND in the presence of either α-synF or α-synR. We did not evidence any major differences between these conditions (Fig. 2*C*,*D*; decrease of 4% for α-synF and 5% for α-synR).

We then measured four parameters: the curvilinear velocity of each moving phase, its run length, the duration of the pauses and the pausing frequency. The velocity (Fig. 2*E*) and run length (Fig. 2*F*) increases (velocity: 31% and 38%; run length: 80% and 100%, for α-synF and R, respectively), the pausing time decreases (Fig. 2*G*; 40% decrease for both fibrillar assemblies), while the pausing frequency increases (Fig. 2*H*; 19% and 25%, for α-synF and R, respectively). These results are summarized in Extended Data Figure 2-2*A*.

**Figure 3.**
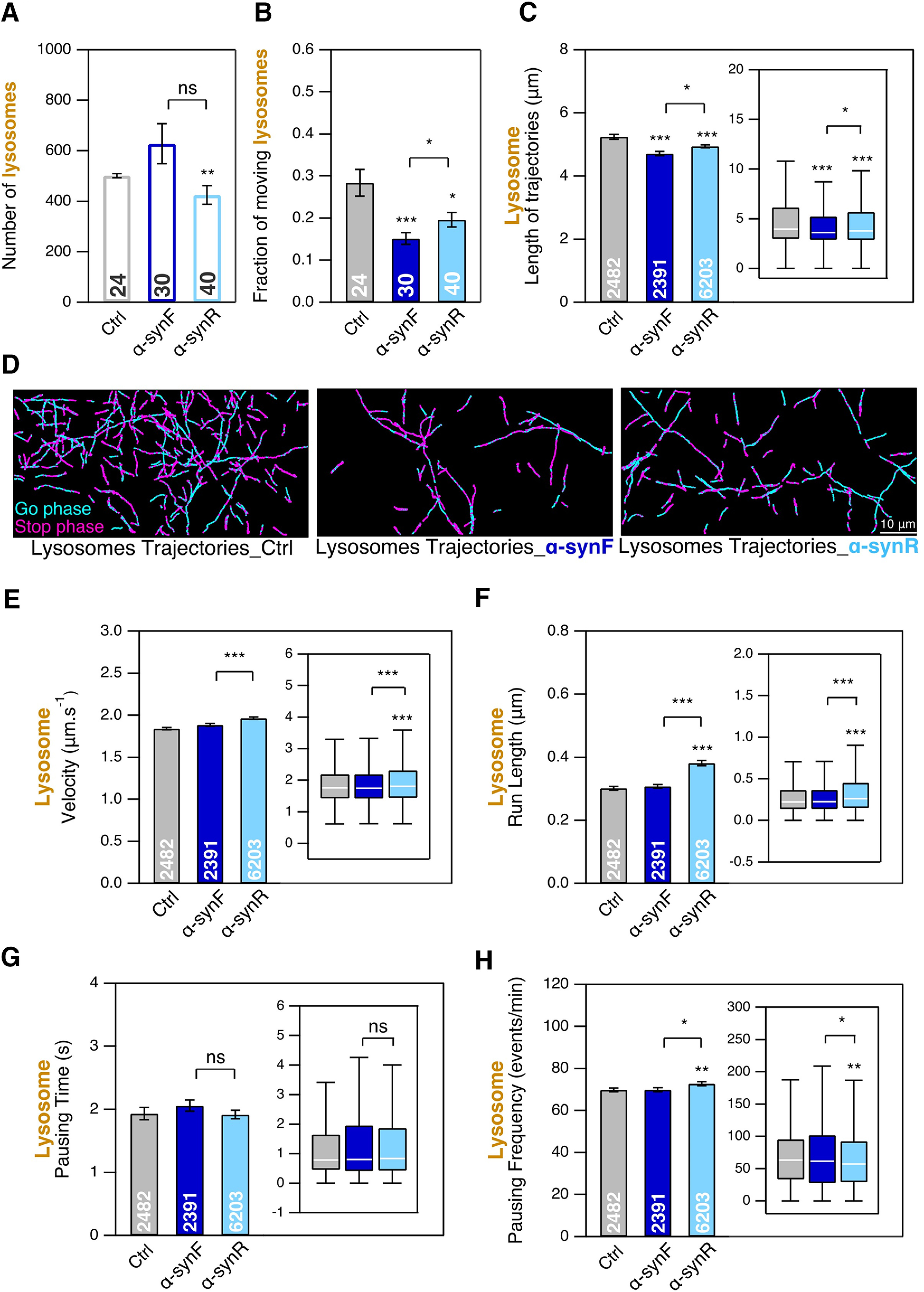
Effect of α-synF or α-synR on the mobility of LysoTracker-labeled lysosomes and their transport in mouse cortical neurons at DIC21; 24-h exposure to α-synF or α-synR at 0.2 μm concentration, compared with nothing added control (Ctrl). ***A***, Number of lysosomes detected per FoV of 40 × 80 μm size during 2 min of observation. ***B***, Fraction of lysosomes having a directed motion. ***C***, Length of lysosome trajectories. ***D***, Examples of lysosome trajectories. Scale bar: 10 μm. ***E*–*H***, Comparison of four transport parameters: curvilinear velocity (***E***), run length (***F***), pausing time (***G***), and pausing frequency (***H***). The number within each bar represents the total number of FoVs (***A***, ***B***) or trajectories (***C***, ***E***–***H***) analyzed from *n *=* *2 coverslips (from one culture). Insets, Box-plots representation of the same dataset. *, ** and *** mean *p*<0.05, *p*<0.01 and *p*<0.001 respectively; ns: non significant.

We next analyzed the same parameters for lysosomes labeled with LysoTracker red, an established marker of lysosomes, with the difference, compared with FND, that all the fluorescent spots, including the static ones, correspond to lysosomes because LysoTracker only become fluorescent once inside lysosomes. α-synR treatment induces a slight but significant decrease (15%) in the number of lysosomes per FoV, while α-synF does not (Fig. 3*A*). This result suggests that α-synR reduce the endocytosis. It is consistent with the observed decrease of FND interacting with neuron (Fig. 2*A*) that possibly reveals their reduced uptake. Furthermore, like for endosomes (Fig. 2*B*), α-synF and R induce a 46% and 32% decrease, respectively, in the fraction of lysosomes having a directed motion (Fig. 3*B*). Analysis of lysosome trajectory lengths indicates a slight decrease (Fig. 3*C*; 9% and 3% for α-synF and R, respectively). Example of FoV showing lysosome trajectories in the different conditions are shown in Figure 3*D*, where the large decrease in the fraction of lysosomes having a directed motion can be clearly seen.

We quantified the same transport parameters for lysosomes than for FND, using the same experimental paradigm. In contrast to what we observed for endosomes transport, exposure of neurons to α-synF did not lead to any changes in lysosomes transport parameters (Fig. 3*E–H*). Interestingly, however, in cortical neurons exposed to α-synR, we measured a slight increase of 6% in lysosomes velocity (Fig. 3*E*) and larger one of 26% in run length (Fig. 3*F*), no significant change in pausing time (Fig. 3*G*), and a slght increase of 4% in pausing frequency (Fig. 3*H*). These results are summarized in Extended Data Figure 2-2*B*.

### Aβ assemblies affect the number of cargoes transported along microtubules without major changes in trajectory length

**Figure 4.**
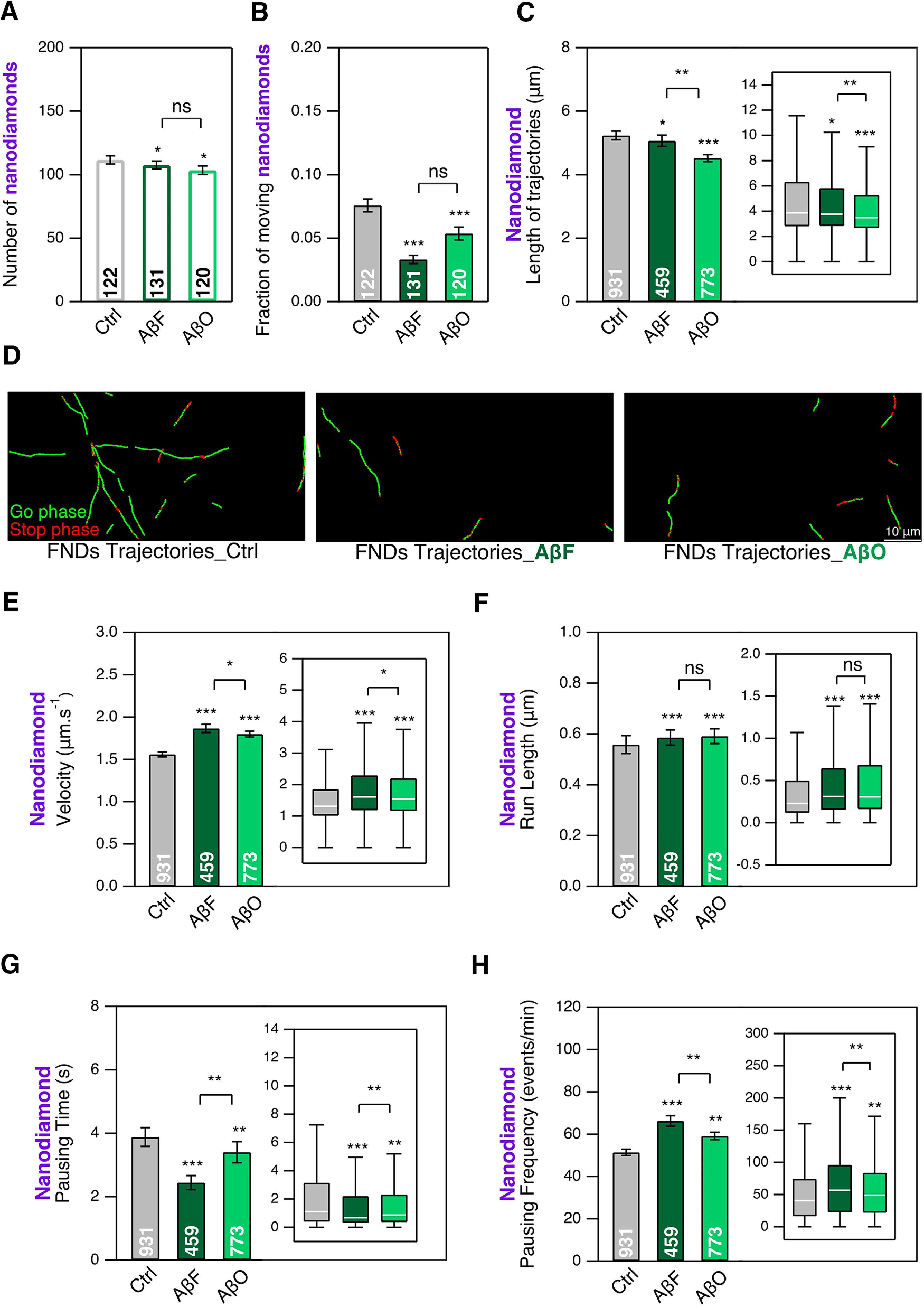
Effect of AβF and AβO on the mobility of endosomes and their transport as measured by tracking FND-containing cargoes in mouse cortical neurons at DIC21; 24-h exposure to AβF and AβO at 1 μm concentration, compared with nothing added control (Ctrl). ***A***, Number of FNDs detected per FoV of 40 × 80 μm size during 2 min of observation. ***B***, Fraction of FNDs-containing cargoes having a directed motion. ***C***, Length of FND trajectories. ***D***, Examples of FND trajectories. Scale bar: 10 μm. ***E*–*H***, Comparison of four transport parameters: curvilinear velocity (***E***), run length (***F***), pausing time (***G***), and pausing frequency (***H***). The number inside the bar represents the total number of FoVs (***A***, ***B***) or trajectories (***C***, ***E*–*H***) analyzed from *n *=* *6 coverslips (3 independent cultures). Insets, Box-plots representation of the same dataset. *, ** and *** mean *p*<0.05, *p*<0.01 and *p*<0.001 respectively; ns: non significant. See also Extended Data Figure 4-1.

We also analyzed the same parameters after mouse cortical neurons exposure to either AβF or AβO at DIC20, for 24 h, followed by intracellular transport measurement at DIC21. We used the common 1 μm concentration that has been reported to have a biological impact (Marshall et al., 2020). Figure 4*A* shows a slight decrease (3% and 7% for AβF and AβO, respectively) of FND interacting with neurons exposed to AβF or AβO, accompanied by a much larger decrease (56% and 29% for AβF and AβO, respectively) of the fraction having directed motions (Fig. 4*B*). FND trajectory lengths stay almost the same for AβF (3% decrease) but are reduced by AβO (Fig. 4*C*,*D*; 13% decrease). We also investigated the effect of Aβ assemblies at the smaller concentration of 0.2 μm, identical to the one of α-syn assemblies. Even at this lower concentration, we could detect for both AβF and AβO small decreases (19% and 18% for AβF and AβO, respectively) in the number of FND per FoV (Extended Data Fig. 4-1*A*) and in the fraction of FND having a directed motion (Extended Data Fig. 4-1*B*; 11% and 18% for AβF and AβO, respectively). To summarize, as for α-syn assemblies (Fig. 3), the exposure of cortical neurons to Aβ assemblies induce important and significant decreases of the endosomal transport.

10.1523/ENEURO.0227-21.2022.f4-1 Extended Data Figure 4-1 Impact of Aβ fibrils and oligomers at the concentration of 0.2 μm on the number of nanodiamonds interacting with cells (A), on the fraction of moving nanodiamonds (B) and on the FND-labeled endosome transport parameters (C–F). Download Figure 4-1, TIF file.

We then measured more precisely the impact of AβF and AβO on endosomal transports parameters. We observed an increase of FND velocity (Fig. 4*E*; 20% and 15% for AβF and AβO, respectively) and run length (Fig. 4*F*; 5% and 7% for AβF and AβO, respectively), a decrease in their pausing time (Fig. 4*G*; 36% and 12% for AβF and AβO, respectively) and an increase of the pausing frequency (Fig. 4*H*; 28% and 15% for AβF and AβO, respectively), with effects more pronounced for AβF than for AβO. Interestingly, the same trends of changes were also observed at the lower AβF and AβO concentration of 0.2 μm (Extended Data Fig. 4-1*C–F*). Let us finally point out that for AβF, the important changes of some transport parameters overall combine in an only very slight decrease in trajectory length as shown in Figure 4*C*, which makes the detailed quantitative analysis performed all the more useful. These results are summarized in Extended Data Figure 2-2*A*.

**Figure 5.**
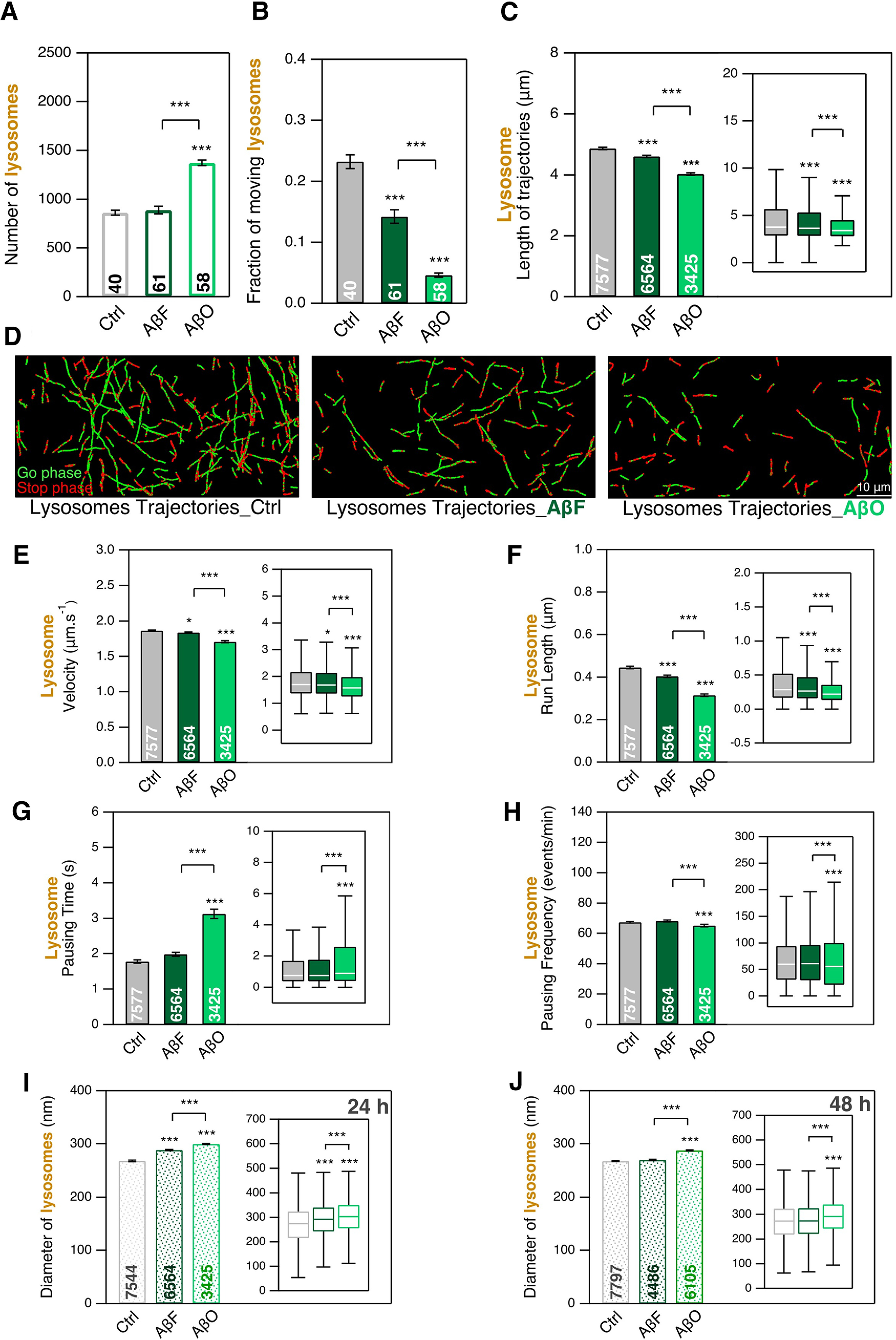
Effect of AβF and AβO on the mobility of LysoTracker-labeled lysosomes and their transport in mouse cortical neurons at DIC21; 24-h exposure to AβF and AβO at 1 μm concentration, compared with nothing added control. ***A***, Number of lysosomes detected per FoV of 40 × 80 μm size during 2 min of observation. ***B***, Fraction of lysosomes having a directed motion. ***C***, Length of lysosome trajectories. ***D***, Examples of lysosome trajectories. Scale bar: 10 μm. ***E–H***, Comparison of four transport parameters: curvilinear velocity (***E***), run length (***F***), pausing time (***G***), and pausing frequency (***H***). ***I***, ***J***, Comparison of lysosome size. The number inside the bar represents the total number of FoVs (***A***, ***B***), trajectories (***E–H***), and lysosomes (***I***, ***J***) analyzed from *n *=* *2 coverslips (from one culture). Insets, Box-plots representation of the same dataset. * and *** mean *p*<0.05 and *p*<0.001 respectively. See also Extended Data Figures 5-1, 5-2, 5-3.

Regarding the impact of Aβ on lysosomal transport, we noticed large differences between the two types of assemblies AβF and AβO. In neurons exposed to AβO, the total number of lysosomes detected in a FoV as compared with controls increased by 50% (Fig. 5*A*) while it stayed unchanged in case of exposure to AβF. The intracellular transport measurements we performed showed that the fraction of moving lysosomes decreased by 1.6-fold and 5.7-fold in neurons exposed to AβF and AβO, respectively (Fig. 5*B*; from 23% for control to 14% for AβF and 4.5% for AβO). Lysosome trajectory lengths were only slightly decreased for AβF (5%) and more significantly reduced for AβO (Fig. 5*C*,*D*; 17% decrease) exposure.

10.1523/ENEURO.0227-21.2022.f5-1 Extended Data Figure 5-1 LysoTracker-labeled compartments size in control (A) and 24-h exposure of Aβ fibrils (B) and oligomers (C) at concentration of 1 μm. Scale bar: 2 μm. Download Figure 5-1, TIF file.

10.1523/ENEURO.0227-21.2022.f5-2 Extended Data Figure 5-2 Effect of α-synF on the mobility of Magic Red-labeled lysosomes and their transport in mouse cortical neurons at DIC21, after 24-h exposure to α-synF at concentration of 0.2 μm, compared to nothing added control. A, Number of lysosomes detected per FoV of 40 × 80 μm size during 2 min of observation. B, Fraction of lysosomes having a directed motion. C, Trajectory length. D, Examples of lysosome trajectories. Scale bar: 10 μm. E–H, Comparison of four transport parameters: curvilinear velocity (E), run length (F), pausing time (G), and pausing frequency (H). I, Diameter of Magic Red-labeled lysosomes. General remarks: the number inside the bar represents the total number of FoVs (A, B) and trajectories (E–I). Inset, Box-plots representations of the same datasets (C–I). Download Figure 5-2, TIF file.

10.1523/ENEURO.0227-21.2022.f5-3 Extended Data Figure 5-3 Effect of AβF on the mobility of Magic Red-labeled lysosomes and their transport in mouse cortical neurons at DIC21 after 24-h exposure to AβF at concentration of 1 μm, compared to nothing added control. A, Number of lysosomes detected per FoV of 40 × 80 μm size during 2 min of observation. B, Fraction of lysosomes having a directed motion. C, Trajectory length. D, Examples of lysosome trajectories. Scale bar: 10 μm. E–H, Comparison of four transport parameters: curvilinear velocity (E), run length (F), pausing time (G), and pausing frequency (H). I, Comparison of Magic Red-labeled lysosome size. General remarks: the number inside the bar represents the total number of FoVs (A, B) and trajectories (E–I). Inset, Box-plots representation of the same dataset. Download Figure 5-3, TIF file.

We also measured lysosome transport parameters in the presence of 1 μm AβF or AβO (Fig. 5*E–H*). For AβF, we observed almost no change in velocity (Fig. 5*E*), pausing time (Fig. 5*G*), and pausing frequency (Fig. 5*H*). In contrast, exposure to AβO, led to a 1.7-fold increase of the pausing time. The run length decreased significantly for both assemblies (Fig. 5*F*; with 9% and 13% decrease for AβF and AβO exposure, respectively). These results are summarized in Extended Data Figure 2-2*B*.

Furthermore, as changes in lysosome size was described in APP mouse transgenic model of Alzheimer’s disease (Gowrishankar et al., 2015), we asked whether detectable changes in lysosome diameter can be quantified on 24- or 48-h exposure to 1 μm AβF and AβO. We detected a slight increase (7%) of lysosome diameter on exposure of neurons to AβF at 24 h that disappears at 48 h. This contrasts with the increase we observed at both time points (11% and 7% at 24 and 48 h, respectively) in neurons exposed to 1 μm AβO (Fig. 5*I*,*J*; Extended Data Fig. 5-1*A–C*). The finding that AβO addition triggers an increase in lysosomes number and size and a decrease in lysosome movements is in agreement with previous reports (Gowrishankar et al., 2015; Marshall et al., 2020).

Finally, as LysoTracker can also label acidic compartments other than lysosomes, in particular late endosomes, we repeated the transport experiment with Magic Red substrate that reveals by fluorescence the Cathepsin B protease activity, taking place more specifically in lysosomes. We observed that for both α-synF (Extended Data Fig. 5-2) and AβF (Extended Data Fig. 5-3) Magic Red-labeled compartments (lysosomes) behaved the same as LysoTracker-labeled ones for all parameters, with in particular a ≈40% decrease of the mobile fraction of Magic Red and LysoTracker-labeled compartment in the presence of the fibrillar assemblies. These results indicates that, in our case, LysoTracker and Magic Red labeling largely overlap and that we can rely on LysoTracker puncta density to quantify endocytic activity as we did. These results are summarized in Extended Data Figure 2-2*C*.

### Transport of α-syn and Aβ assemblies within cortical neurons

We also assessed α-syn and Aβ assemblies transport within cortical neurons while documenting their impact on endosomes and lysosomes dynamics. As ATTO 488 dye used to label α-syn and Aβ assemblies exhibit no emission spectrum overlap with neither FND nor LysoTracker deep red, we were able to measure simultaneously the transport properties of endosome or lysosome and the assemblies on two-color channels.

**Figure 6.**
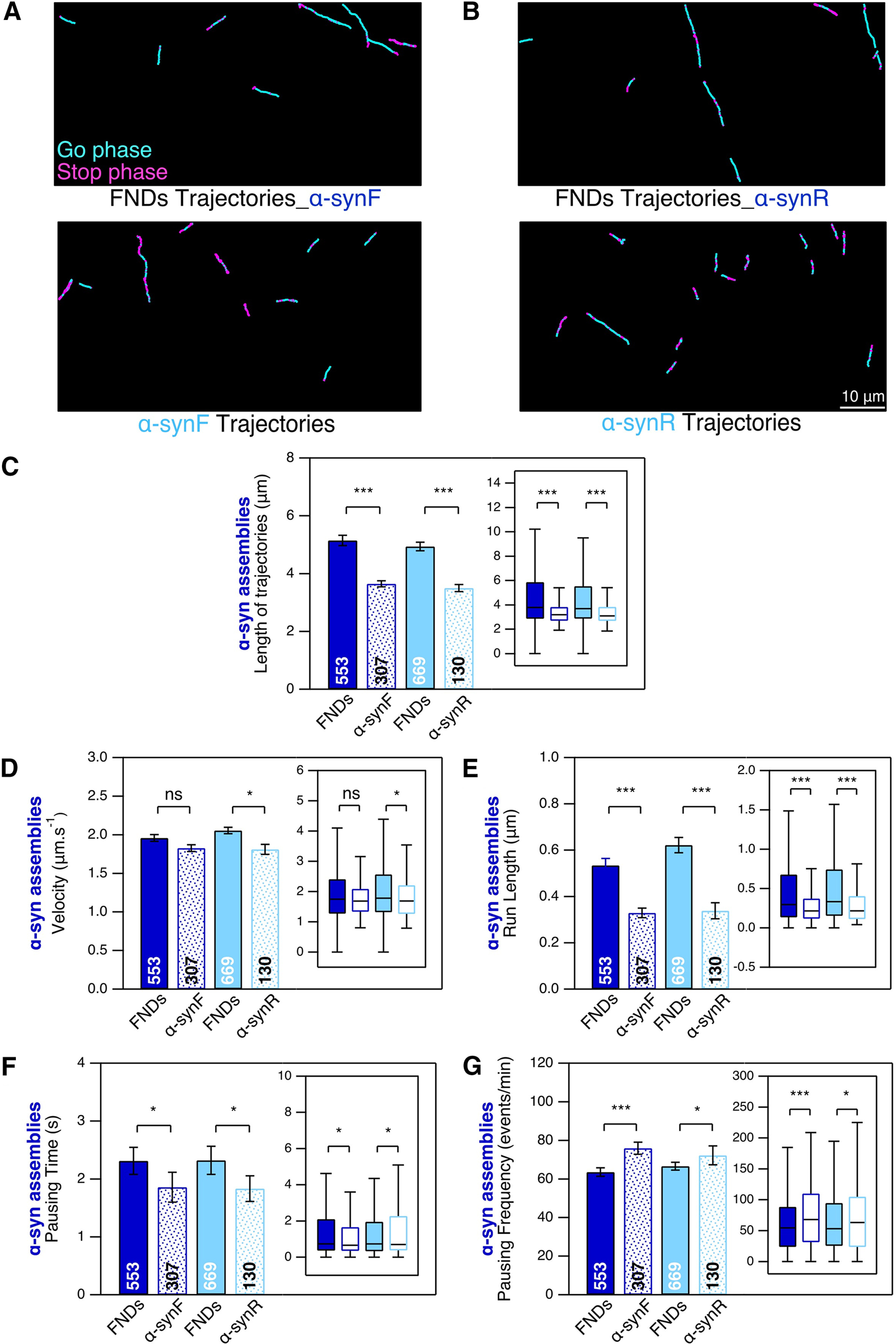
Intraneuronal transport of ATTO 488-labeled α-synF and R in mouse cortical neurons at DIC21. DIC20 cortical neurons were exposed to α-synF and R during 24 h, at concentration of 0.2 μm. ***A***, ***B***, Examples of α-synF and R, and FND trajectories (in the presence of α-synF and R). Scale bar: 10 μm**. *C***, Length of α-synF, α-synR, and FND trajectories. ***D*–*G***, Comparison of four transport parameters: curvilinear velocity (***D***), run length (***E***), pausing time (***F***), and pausing frequency (***G***). The number inside the bar represents the total number of trajectories analyzed from *n *=* *8 coverslips (4 independent cultures) is indicated in each bar. Insets, Box-plots representation of the same dataset. * and *** mean *p*<0.05 and *p*<0.001 respectively; ns: non significant.

We first studied α-syn fibrillar assemblies transport (Fig. 6). We found that α-synF and R display directed movements as shown by examples of trajectories in Figure 6*A*,*B*. We compared these motions to the endosomal transport in the presence of α-syn fibrillar polymorphs. Interestingly, α-syn F and R trajectories are ∼29% shorter than those of FND (Fig. 6*C*). We also compared the transport parameters and found smaller velocity (Fig. 6*D*; 5% and 10% for α-synF and α-synR, respectively), run length (Fig. 6*E*; 39% and 46% for α-synF and α-synR, respectively), and pausing time (Fig. 6*F*; 19% and 21% for α-synF and α-synR, respectively) for α-synF and α-synR-loaded cargoes compared with those of FND-containing endosomes. On the contrary, α-synF and R pausing frequencies were larger than those of FND (Fig. 6*G*; 19% and 8% for α-synF and α-synR, respectively). The shorter trajectories length and run-length together with the larger pausing frequency, suggest that cargoes loaded with α-synF- and R are transported less efficiently than those containing FND.

**Figure 7.**
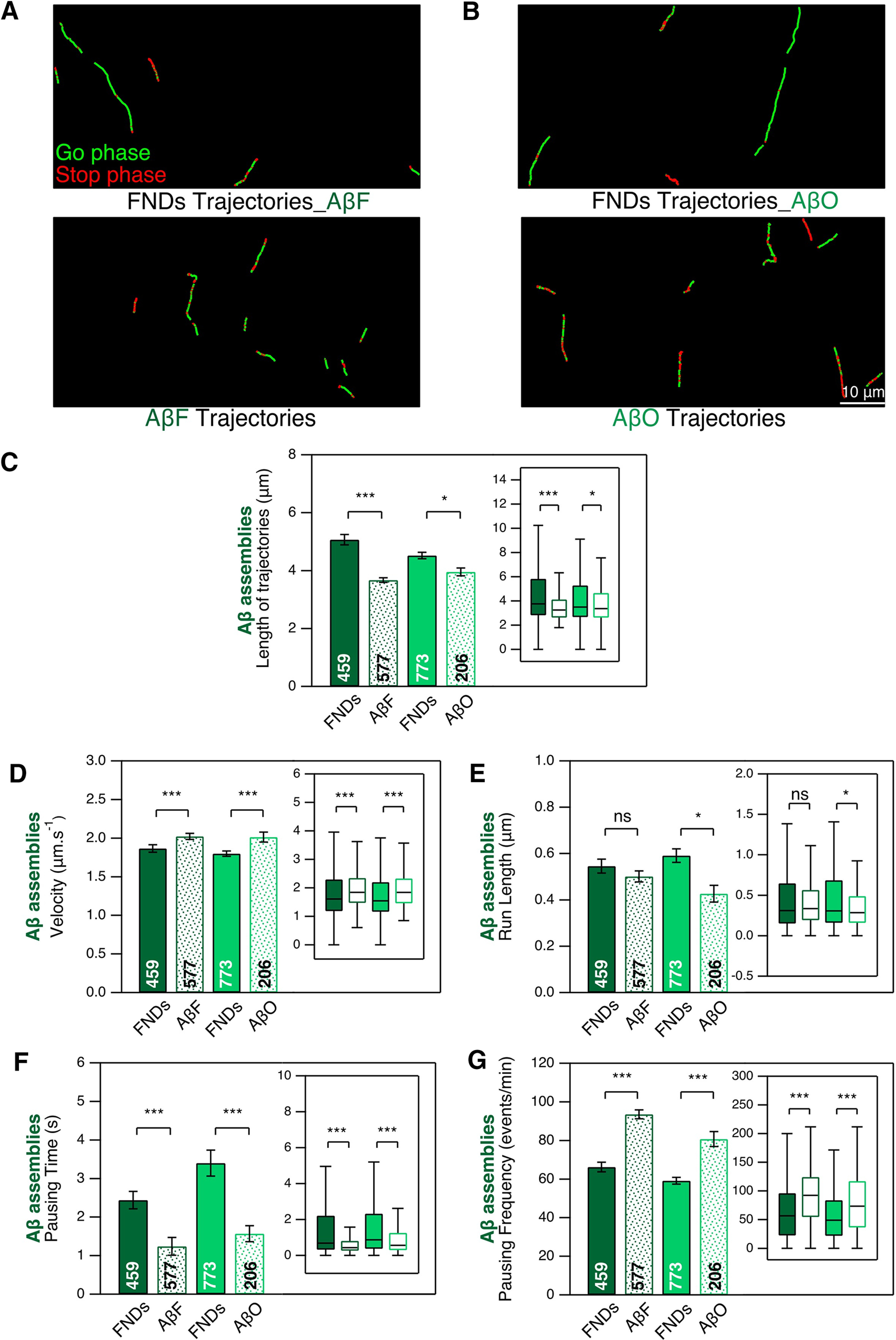
Intraneuronal transport of ATTO 488-labeled AβF and AβO in mouse cortical neurons at DIC21. DIC20 cortical neurons were exposed to AβF and AβO (1 μm) during 24 h. ***A***, ***B***, Examples of trajectories. Scale bar: 10 μm. ***C***, Length of AβF, AβO and FND trajectories. ***D*–*G***, Comparison of four transport parameters: curvilinear velocity (***D***), run length (***E***), pausing time (***F***), and pausing frequency (***G***). The number inside the bar represents the total number of trajectories analyzed from *n *=* *6 coverslips (from 3 independent cultures) is indicated in each bar. Insets: box plots representation of the same dataset. * and *** mean *p*<0.05 and *p*<0.001 respectively; ns: non significant. See also Extended Data Figure 7-1.

Similarly, we also investigated ATTO 488-labeled AβF and AβO intraneuronal transport (Fig. 7) at the concentration of 1 μm, as we could not detect their fluorescence signal at 0.2 μm. Both species exhibit directed transport as shown by examples of trajectories in Figure 7*A*,*B*. These trajectories are shorter than the ones of FND in the same conditions (Fig. 7*C*), as for α-syn fibrillar assemblies. Regarding the transport parameters, compared with FND, AβF, and AβO have slightly larger velocity (Fig. 7*D*; 8% and 12% for AβF and AβO, respectively), and a trend toward a shorter run-length (Fig. 7*E*; 7% and 28% for AβF and AβO, respectively). As for α-syn fibrillar assemblies, AβF and AβO exhibit a much shorter pausing time (Fig. 7*F*; 49% and 53% for AβF and AβO, respectively) and a much larger pausing frequency (Fig. 7*G*; 41% and 36% for AβF and AβO, respectively). The shorter trajectories length and run-length together with the larger pausing frequency suggest that, like in the case of α-syn, cargoes loaded with AβF and AβO are transported less efficiently than those containing FND.

10.1523/ENEURO.0227-21.2022.f7-1 Extended Data Figure 7-1 Number of AβF and AβO trajectories detected per FoV of 40 × 80 μm during 2 min of observation. Cortical neurons were exposure to AβF and AβO (1 μm) during 24 h (A) and 48 h (B). The number inside the bar represents the total number of FoVs analyzed from n = 6 coverslips (from three cultures) for 24 h and n = 2 coverslips (from one culture). Download Figure 7-1, TIF file.

Moreover, since differences in AβO and AβF uptake in cultured neurons were recently reported (Vadukul et al., 2020), we also investigated the related aspect of the number of Aβ assembly trajectories per FoV at 24- and 48-h time points (Extended Data Fig. 7-1). We did not observe differences in the number of trajectories 24 h after addition of the assemblies (Extended Data Fig. 7-1*A*) in agreement with (Vadukul et al., 2020); see their Figure 4B. However, at 48-h time point, we measured a ≈2.5 lower number of AβO trajectories compared with AβF ones (Extended Data Fig. 7-1*B*), which differs to uptake results of (Vadukul et al., 2020) at 72-h time point, who reported a ≈1.5 times larger amount of internalized AβO compared with AβF (sonicated). Our observations differ from (Vadukul et al., 2020), but several reasons may explain this discrepancy: (1) we quantify only the moving fraction of Aβ assemblies; (2) we do not have the 72-h point; and finally (3) we do not use the exact same AβF.

**Figure 8.**
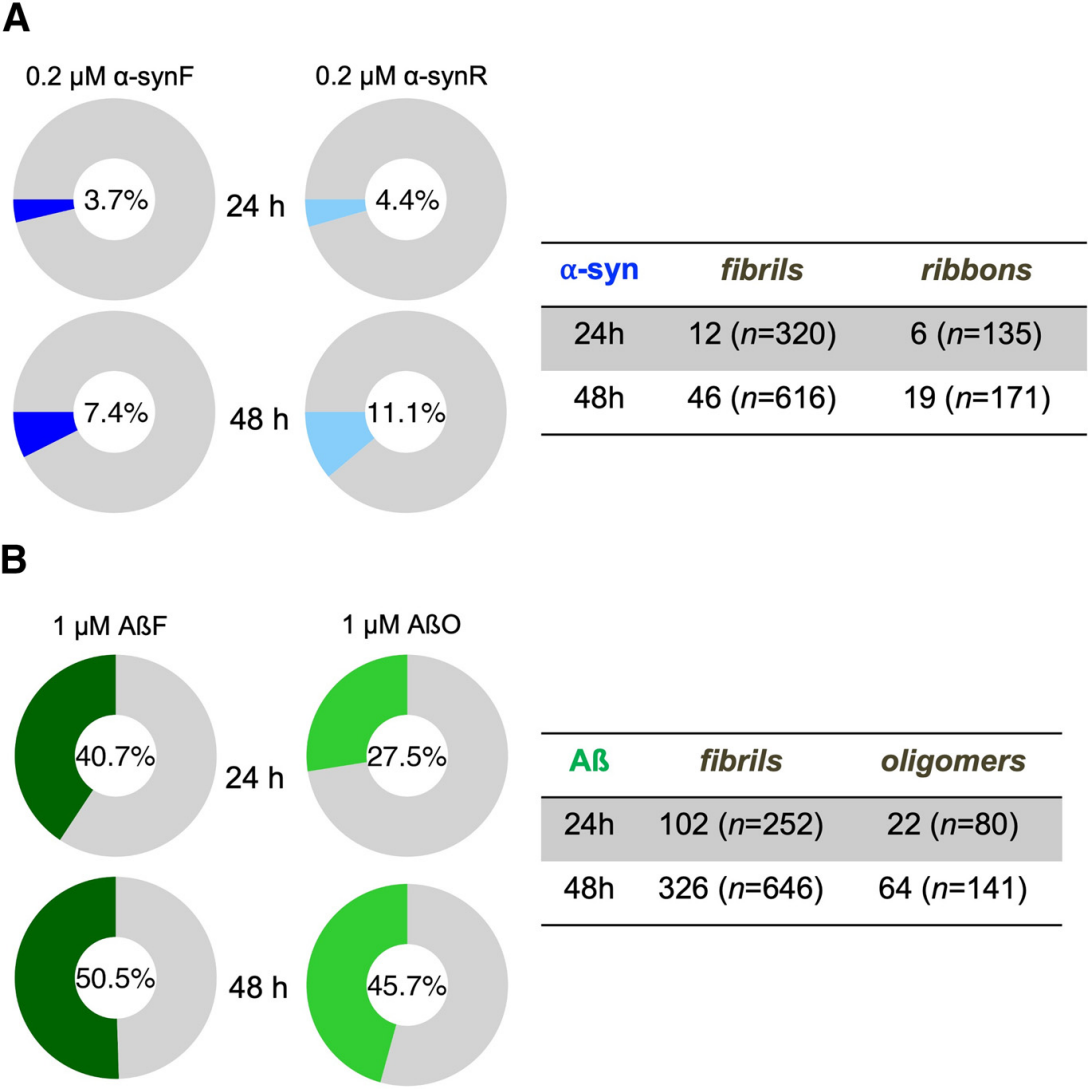
Colocalized events between moving neurodegenerative disease-related molecular species and moving lysosomes at different time points. ***A***, α-synF and α-synR were incubated for 24 and 48 h, at concentration of 0.2 μm. ***B***, AβF and AβO were incubated for 24 and 48 h, at concentration of 1 μm. The number inside the donut plot represents the percentage of moving α-syn or Aβ assemblies colocalized with lysosomes (LysoTracker labeled). The table on the right panel indicates the number of neurodegenerative-related molecular species trajectories colocalized with lysosomes. *n* represents the total number of trajectories. The percentage and number of trajectories in each time point were analyzed from two coverslips (from one culture). See also Extended Data Figure 8-1.

Finally, we assessed in a quantitative manner the co-localization of α-syn and Aβ assemblies with lysosomes, as a function of neuron exposure time at 24 and 48 h (Fig. 8). While the fraction of α-syn fibrillar assemblies moving within lysosomes increases from ≈4% at 24 h to 12–14% at 72 h (Fig. 8*A*), it reaches already ≈41% at 24 h for Aβ fibrils continuing its increase up to ≈51% at 48 h (Fig. 8*B*). AβO moving in or with lysosomes have slightly lower colocalization proportions, however much larger than for α-syn fibrillar assemblies. We repeated these analyses with Magic Red labeling instead of LysoTracker for α-synF (Extended Data Fig. 8-1*A*) and AβF (Extended Data Fig. 8-1*B*) at 24 h and achieved similar results, with a ≈40% colocalization fraction of AβF with Magic Red-labeled lysosomes, and only ≈7% for α-synF.

10.1523/ENEURO.0227-21.2022.f8-1 Extended Data Figure 8-1 Fraction of protein assemblies, α-synF (A) or AβF (B) trajectories colocalizing with Magic Red puncta trajectories, as inferred from the tables indicating the number of protein assembly trajectories colocalizing with Magic Red puncta and the total number of assembly trajectories in parenthesis. Data from two coverslips and one culture in both α-synF and Aβ cases. Download Figure 8-1, TIF file.

## Discussion

In this work, we investigated the generic effects of α-syn fibrillar polymorphs (fibrils and ribbons) and Aβ assemblies (oligomers and fibrils) on endosomal and lysosomal movements in mouse cortical neurons. In neurons, early endosomes and lysosomes move using different types of machinery. Also, the former are compartmentalized (dendrites vs axon) while the latter are not (Winckler et al., 2018). However, because of the high density of the cultures it was not possible to identify unambiguously the compartment (dendrite or axon) in which the traced vesicles moved, and therefore we could not study separately the impact of the protein assemblies on axonal and dendritic endolysosomal transports.

### Potential consequences of a decrease in the number of cargoes transported at a given time within cortical neurons

The exposure of cortical neurons to α-syn and Aβ assemblies led to important reductions (between 32% and 56%) of moving endosomes and lysosomes along neuronal branches (Figs. 1, 2). We previously showed that pathogenic α-syn and Aβ assemblies bind the plasma membrane with, as a consequence, a redistribution of essential membrane proteins (Renner et al., 2010; Shrivastava et al., 2013, 2015). We further reviewed the physico-pathogenic mechanisms at the origin and resulting from pathogenic proteins assemblies-plasma membrane components interactions (Shrivastava et al., 2017). The reduction we report here might result from changes in membrane dynamics and endocytosis rate.

Exposure of cortical neurons to α-syn and Aβ assemblies affected moving FND-containing endosome properties. α-Syn fibrillar assemblies increased their velocity by 31–38% and run length by 80–100% (Fig. 2*E*,*F*) and decreased their pausing time by 40% (Fig. 2*G*) while increasing their pausing frequency to a lesser extend (19–25%; Fig. 2*H*). We observed similar effects but less pronounced for moving FND-containing endosomes when neurons are exposed to AβF or AβO (Fig. 4*E–H*). These changes reflect an increase in mobility of moving FND-containing endosomes. Hence, while decreasing the fraction of moving FND-labeled endosomes, α-synF and R or AβF and AβO, increase the overall mobility of the moving ones (Extended Data Fig. 2-2*A*).

A decrease in the number of moving endosomes or lysosomes can affect protein quality control, accompanied by limited elimination of damaged membrane and cytosolic proteins, protein aggregates, and membranous organelles (Winckler et al., 2018). Furthermore, considering that lysosomes and late endosomes act as mRNA translation platforms (Cioni et al., 2019; Liao et al., 2019; Fernandopulle et al., 2021), changes in the number of cargoes transported at a given time within a cortical neuron is expected to dramatically impact mRNA translation platform either in dendrites or in axons. In particular, the regulation of protein synthesis and degradation at the neuronal synapse is local and dynamic and modify the synaptic proteome autonomously during plasticity (Giandomenico et al., 2022). Hence, the synaptic function can be impacted if the number of moving lysosomes is affected.

### Effect of α-syn and Aβ assemblies on lysosomes transport

We observed that α-synF (Fig. 3*E–H*) and AβF (Fig. 5*E–H*) barely impact the lysosomal transport parameters as compared with the control. In contrast, α-synR (Fig. 3*E–H*) and AβO (Fig. 5*E–H*) induce significant changes of lysosomes transport parameters (Extended Data Fig. 2-2*B*). Furthermore, the size of lysosomes significantly increased in the presence of AβO (Fig. 5*I*,*J*). This set of impairments of lysosomal transport in Alzheimer-related context are in full agreement with previous reports (Gowrishankar et al., 2015; Marshall et al., 2020). Indeed, using a mouse model of Alzheimer’s disease (Gowrishankar et al., 2015) evidenced axonal lysosome accumulations with local impairment in the retrograde axonal transport of lysosome precursors. Similarly, Marshall et al. (2020) found that misfolded Aβ_42_ impacts the endo-lysosomal pathway. They reported impairments in the uptake of proteins that use a dynamin-dependent endosomal mechanism and accumulation of lysosomes.

### Intraneuronal transport of neurodegenerative-related molecular species

We were able to quantity the intraneuronal transport parameters for α-syn and Aβ pathogenic species (Figs. 6, 7). We found that cargoes loaded with α-synF and R exhibit a more dynamical transport compared with those containing FND that are characterized by a larger pausing frequency and shorter run-length and pausing time (Fig. 6*D–G*). AβF and AβO-containing endosomes exhibit similar characteristics (Fig. 7*D–G*) with in addition an increase in velocity, not observed for α-synF and R. These results suggest that similar molecular mechanisms are at play in the transport of the two α-syn fibrillar polymorphs, the AβF and AβO. However, the ≈7-fold larger fraction of AβF and AβO found in moving lysosomes compared with α-syn assemblies (Fig. 8), also indicate differences in the molecular interactions of Aβ assemblies with lysosomes. These results suggest a cellular triage leading to differential transport of α-syn and Aβ assemblies.

### Potential application in drug discovery assay

We report here the use of a model based on primary mouse cortical neurons where it is possible to identify a robust decrease in the number of vesicles moving intracellularly (Figs. 2*B*, 3*B*, 4*B*, 5*B*), in the order of 30–50%, and up to 5-fold for lysosomes in neurons exposed to AβO (Fig. 5*B*). Such a large decrease is likely to impact the physiology of neurons and it is reasonable to consider that the transport of other cargoes, such as mitochondria and RNA granules, is also affected. This endolysosomal transport impairment endophenotype can be instrumental in generating large-scale drug-discovery campaigns (i.e., >10^5^ compounds) as used in more complex human cellular models (Park et al., 2021).

The cargoes transport blockade within cortical neurons we report could either result from a direct interaction between pathogenic aggregates and the intraneuronal transport machinery or a pathogenic aggregates-mediated transcriptional changes in transport proteins expression (Lee et al., 2004; Encalada and Goldstein, 2014; Guo et al., 2020). Analysis of neuronal immuno-precipitates of α-syn fibrillar assemblies and Aβ polymorphs may be instrumental to identify the molecular partners that directly interact with these protein assemblies. A recent postmortem proteomics study identified proteins whose abundance changed at different stages of Alzheimer’s disease (Li et al., 2021). At its early stage, differentially expressed proteins of “clathrin‐coated endocytic vesicle membrane” (GO: 0030669) and the secretory pathway (R‐HSA‐432720: “Lysosome Vesicle Biogenesis” and R‐HSA‐432722: “Golgi Associated Vesicle Biogenesis”) classes were over‐represented. Comparison of proteome profile changes in our neuronal model with (Li et al., 2021) profiles can be instrumental to identify druggable targets to enhance the number of transported cargoes.

Finally, we quantified intraneuronal transport of neurodegenerative-linked molecular species (α-syn fibrillar polymorphs, AβF, and oligomers) whose transport characteristics are distinct from those of endosomes but for which no molecular characterization is yet available. Furthermore, we observed that all these protein assemblies are transported intracellularly in cortical neurons with very similar quantitative characteristics. We could not detect differences in their transport parameters. Further work will be required to identify a possible common transport mechanism and the specific molecules involved in this transport. This identification can then be harnessed to selectively inhibit this transport.

These results also need to be considered from the standpoint of the prion-like spread of pathogenic protein particles between neurons (Brundin et al., 2010; Hardy and Revesz, 2012; Jucker and Walker, 2018). Selective inhibition may avoid the spread of these neurotoxic species. Thus, advances in the identification of targets involved in cargoes loaded with pathogenic protein aggregates transport may lead to novel neuroprotective therapeutic avenues.

## Synthesis

Reviewing Editor: Karl Herrup, University of Pittsburgh School of Medicine Decisions are customarily a result of the Reviewing Editor and the peer reviewers coming together and discussing their recommendations until a consensus is reached. When revisions are invited, a fact-based synthesis statement explaining their decision and outlining what is needed to prepare a revision will be listed below. The following reviewer(s) agreed to reveal their identity: Louise Serpell. Note: If this manuscript was transferred from JNeurosci and a decision was made to accept the manuscript without peer review, a brief statement to this effect will instead be what is listed below.

The authors have been responsive to the concerns raised and have done a good job on the revision. The only remaining concern would be to polish the language a bit. As only one example, the first sentence of the revised text concerning the response to Point 4 reads:

“In order to determine if this important reduction of FND moving fraction is related to the binding of the protein assemblies to the neuronal branches membrane, possibly leading to a reduced endocytosis, we tried in the case of ⍺-synF to wash away the aggregates just before the addition of FND (Materials and Methods), but we found that the interaction of the nanodiamonds with neuron did not change in washed- neuron condition compared to unwashed (Suppl. Fig. S1A-C); in particular, we did not observe differences in the fraction of moving FND.”

It would be much easier to read if this were broken up into shorter sentences as for example:

“We wished to determine if the reduction of the FND moving fraction was related to protein assemblies binding to the membranes of the neuronal branches leading to reduced endocytosis. To test this, we washed the ⍺-synF preparations to remove all loosely bound aggregates just before adding the FND (Materials and Methods). We found no noticeable differences in the fraction of moving FND (Suppl. Fig. S1A-C) suggesting that the interactions of the nanodiamonds with the neuron were nearly the same.”

Any number of alternative phrasings would be fine, but before a final acceptance is issued, please go over the text carefully for clarity. The work will not be re-reviewed, but I will look over the changes. Thanks for your help in this matter.

## Author Response

Author’s reply to the review report

We are thankful to the reviewers and to the editor for their positive and constructive comments that led to a significantly improved manuscript.

We added new results based on experiments proposed by reviewers with 8 new extended Figures and modified the previous manuscript accordingly.

Here, we address their suggestions point-by-point and indicate the changes made in the revised manuscript. In particular we added six supplementary Figures.

Comments and suggestions for Authors:

Point 1: The authors could begin by clarifying the extent to which they saw differences between oligomers and fibrils. Previous studies have shown that there is much less uptake of Abeta fibrils than oligomers (see Vadukul et al., FEBS Letter 2020). The relationship of the current findings to these older ones should be discussed.

Reply: Contrary to (Vadukul et al. 2020) we did not quantify the uptake of Aß assemblies as we could not distinguish their precise location, either inside or outside the neuronal branches. However, we added Suppl. Fig. S6 in which we show the quantifications, in field-of-views of size 40 μm×80 μm, of the average number of Aß assemblies fluorescence spots having directed motions (i.e. being inside the neurons) during 2-minutes duration videos. Like for the uptake quantification reported in (Vadukul et al. 2020), we did not observe differences in the number of trajectories 24h after addition of the assemblies. However, at 48h time point, we measured a ≈2.5 lower number of AßO trajectories compared to AßF ones, which differs to uptake results of (Vadukul et al. 2020) at 72 h time point, who report a ≈1.5 times larger amount of internalized AßO compared to AßF.

To account for these complementary analyses and their comparison to data reported in (Vadukul et al. 2020), we added in the subsection “Transport of ⍺-syn and Aß assemblies within cortical neurons,” the following text (page 19, lines 467-478):

"Moreover, since differences in AßO and AßF uptake in cultured neurons were recently reported (Vadukul et al., 2020), we also investigated the related aspect of the number of Aß assembly trajectories per field-of-view at 24 h and 48 h time points (Suppl. Fig. S6) We did not observe differences in the number of trajectories 24 h after addition of the assemblies (Suppl. Fig. S6A) in agreement with Fig. 4B of (Vadukul et al. 2020). However, at 48 h time point, we measured a ≈2.5 lower number of AßO trajectories compared to AßF ones (Suppl. Fig.6B), which differs to uptake results of (Vadukul et al. 2020) at 72 h time point, who reported a ≈1.5 times larger amount of internalized AßO compared to AßF(sonicated). Our observations differ from (Vadukul et al. 2020), but several reasons may explain this discrepancy: (i) we quantify only the moving fraction of Aß assemblies; (ii) we do not have the 72 h point, and finally (iii) we do not use the exact same AßF”.

Point 2: The percentages of FND (from Haziza et al 2017) that co-localized with other endosomal markers don’t add to 100% (31%). Where is the other 69% of FNDs located? This also means that when you follow just the signal for FND, only 31% of that signal localizes to some endosomal compartment which raises the question of what the FND fluorescence is monitoring. Since, FND labeling can tag multiple types of endosomes at the same time, confidence in the interpretation of the data from the FND fluorescence experiments is diminished.

Reply: As we did not address the co-localization of FNDs with the markers of the different types of endosomes in this study, we removed the associated paragraph:

"We previously studied the colocalization of FND with a specific marker of the different endosomal-lysosomal compartments (Fig. 1A) 24 hours after internalization (Haziza et al., 2017). We observed that FND colocalized (fraction indicated) with endosome and lysosome at different stages of their development, each characterized by specific membrane protein markers (Haziza et al., 2017): EEA1(5%) and Rab5 (9%) for early endosome (EE); Rab7 (4.5%) for late endosome; Rab11 (4.5%) for recycling endosome (RE); LysoTracker (5%) for the lysosome and TGN38 (3%) for the trans Golgi network to which EE can partly fuse.”

and we complemented the preceding sentence which became (page 12, line 304-306): “Our incubation protocol was designed to strongly limit any non-specific interactions of FND, like their attachment to the coverslip supporting the culture, and favor their interaction with neuron membranes and their subsequent internalization in endosomes (Haziza et al., 2017).”

Point 3: Early endosomes and lysosomes move around the neuron using different types of machinery. Also, the former are compartmentalized (dendrites vs. axon) while the latter are not. The relatively high density of the cultures and the lack of certainty as to whether measurements of endosome behavior are being made in a dendrite or an axon need more care in presentation.

Reply: We took into account the judicious remark of the referees and added to the beginning of the discussion the following paragraph (page 20, lines 490-498), citing the new reference (Winckler et al., 2018):

"In this work, we investigated the generic effects of ⍺-syn fibrillar polymorphs (fibrils and ribbons) and Aß assemblies (oligomers and fibrils) on endosomal and lysosomal movements in mouse cortical neurons. In neurons, early endosomes and lysosomes move using different types of machinery. Also, the former are compartmentalized (dendrites vs. axon) while the latter are not (Winckler et al., 2018). However, due to the high density of the cultures it was not possible to identify unambiguously the compartment (dendrite or axon) in which the traced vesicles moved, and therefore we could not study separately the impact of the protein assemblies on axonal and dendritic endolysosomal transports.”

Point 4: The authors maintain “that both fibrillar polymorphs impact the FND binding...” but are silent on whether this is an impact on internalization? Has this distinction been made? One possibility is to try an experiment where the external aggregates are washed away before adding the FND.

Reply: Following the referee’s suggestions, we investigated whether replacing the medium several times before adding the FND (see “Washing protocol” added to the Materials and Methods) section), could detach the protein assemblies and impact FND interactions with the neurons. We carried out such an analysis in the case of ⍺-synF and found that the interaction of FND with neuron did not change in washed-neuron condition compared to unwashed (Suppl. Fig. S1A-C); in particular, we did not observe an increase in the fraction of moving FND. We concomitantly measured the amount of ATTO 488-labelled assemblies along the neuronal branches (as quantified by the dye fluorescence) and could not see difference before and after washing (Suppl. Fig. S1D-E), which is in agreement with FND-neuron interaction results, and indicate a strong binding of ⍺-synF to the neuronal membrane. We added the following paragraph to the subsection “⍺-synF and R affect the number of cargos transported along microtubules without major changes in trajectory length” (page 13, line 324-334):

"In order to determine if this important reduction of FND moving fraction is related to the binding of the protein assemblies to the neuronal branches membrane, possibly leading to a reduced endocytosis, we tried in the case of ⍺-synF to wash away the aggregates just before the addition of FND (Materials and Methods), but we found that the interaction of the nanodiamonds with neuron did not change in washed-neuron condition compared to unwashed (Suppl. Fig. S1A-C); in particular, we did not observe differences in the fraction of moving FND. We concomitantly measured the amount of ATTO 488-labelled assemblies along the neuronal branches (as quantified by the dye fluorescence intensity) and could not see any differences before and after washing (Suppl. Fig. S1D-E), which is in agreement with FND-neuron interaction results, and indicate a strong binding of ⍺-synF to the neuronal membrane.”

We also added a description of the washing procedure (page 7, line 182-186) and of the quantification of fluorescence intensity of ATTO 488-labeled ⍺-synF in cortical neuron branches (page 11, lines 277-286) in the Materials and Methods.

Point 5: The authors should be more cautious in their interpretation of the LysoTracker results. Lysotracker accumulates in all acidic compartments - lysosomes but also late endosomes and multivesicular bodies). Also, its fluorescence is independent of pH (in contrast to LysoSensor). Independent methods of labeling lysosomes should be considered such as DQ-BSA, pulse-chase labeled dextran, BODIPYFL-pepstatin A, or MagicRed. In this same vein, the evidence that ⍺-synR reduces the endocytosis is based on the decreased number of LysoTracker puncta, which could reflect changes in other acidic vesicles as well. It would help if additional lysosome markers were used. Decreased LAMP or cathepsin proteins shown by immunoblot would strengthen this assertion.

Reply: Concerning the specificity of LysoTracker, which could also label late endosomes, we followed the reviewer’s suggestion and carried out complementary experiments with an alternative fluorescent marker and selected Magic Red Cathepsin B (ref. ICT937, Bio-Rad) that labels the lysosomes with a higher specificity than the LysoTracker.

We observed that for both ⍺-synF (Suppl. Fig. S2) and AßF (Suppl. Fig. S3) Magic Red-labelled compartments (lysosomes) behaved the same as LysoTracker-labelled ones for all parameters, with in particular a ≈40% decrease of the mobile fraction of Magic Red and LysoTracker-labelled compartment in the presence of the fibrillar assemblies, and similar colocalization data at 24 h, i.e. small colocalization of 4-7% for ⍺-synF compared to 40% for AßF.

To account for these results, we added the following paragraph at the end of the sub-section “Aß assemblies affect the number of cargos transported along microtubules without major changes in trajectory length” (page 17, lines 422-433):

"Finally, as LysoTracker can also label other acidic compartments than lysosomes, in particular late endosomes, we repeated the transport experiment with Magic Red substrate that reveals by fluorescence the Cathepsin B protease activity, taking place more specifically in lysosomes. We observed that for both ⍺-synF (Suppl. Fig. S4) and AßF (Suppl. Fig. S5) Magic Red-labelled compartments (lysosomes) behaved the same as LysoTracker-labelled ones for all parameters, with in particular a ≈40% decrease of the mobile fraction of Magic Red and LysoTracker-labelled compartment in the presence of the fibrillar assemblies, and similar colocalization data at 24 h, i.e. small colocalization of 4-7% for ⍺-synF compared to 40% for AßF. These results indicates that, in our case, LysoTracker and Magic Red labeling largely overlap and that we can rely on LysoTracker puncta density to quantify endocytic activity as we did.”

Point 6: There seems to be an unexplained difference between the data in Fig 4B and Fig S1B, specifically whether the responses depend on the concentration of the different states of amyloid aggregation - oligomers vs. fibrils.

Reply 6: In Fig. S1B at the concentration of 0.2 μM, we display no (for AßO) and very little (for AßF) differences in the fraction of moving endosomes. In contrast, at 1 μM concentration, we found a statistically highly significant reduction of the fraction of moving FND for both AßF and AßO (Fig. 4B) but no difference between the two polymorphs. From this data we can hardly state with confidence that there is a dose-dependent effect of Aß on FND-moving fraction only for oligomers and not for fibrils, which is why we prefer not to add further comments.

Point 7: There are certain changes that could be made for clarity of presentation. For example, pictures of the analysis of the size of lysosomes would help the reader to not only understand the calculations explained in the Methods section, but also to provide the evidence for the claim of the change in size. Also, the pie-chart figure in the supplementary is informative and should be at least considered for inclusion in the main text.

Reply: We followed the reviewer’s suggestions and added the Suppl. Fig. S3 in which we display examples of lysosome diameter measurements (from LysoTracker puncta) in the control, AßF and AßO situation. We also moved the pie-chart figures of the protein assemblies/lysosome colocalization data from supplementary figure to the new main Figure 8.

Point 8: In the Introduction the authors propose to address questions of spread from one neuron to another, but this is not actually addressed by the experiments performed. This should be rephrased appropriately.

Reply: We agree with the reviewer and removed the point “(iii)” of the introductory paragraph that listed the questions addressed, that reduced now to (page 4, line 110-112):

"Our data allow to address two complementary questions: (i) do ⍺-synF, ⍺-synR, AßF and AßO influence the fraction of cargoes moving along the microtubules; (ii) do they impact the dynamics of intracellular endosomal and lysosomal transport.”

Point 9: Lastly, the importance of the effects on endo-lysosomal dynamics could be discussed in more depth. The authors should clarify why this would be important in changing our understanding of the pathways leading to neurodegenerative diseases. What specific detrimental effects would they expect for the impacted neurons?

Reply: We followed the reviewers advices and completed the last paragraph of the sub-paragraph “Potential consequences of a decrease in the number of cargos transported at a given time in a cortical neuron” (page 21, lines 519-529) as follows (with the additional reference (Giandomenico et al., 2021)):

"A decrease in the number of moving endosomes or lysosomes can affect protein quality control, accompanied by limited elimination of damaged membrane and cytosolic proteins, protein aggregates, and membranous organelles (Winckler et al., 2018). Furthermore, considering that lysosomes and late endosomes act as mRNA translation platforms (Cioni et al., 2019; Liao et al., 2019; Fernandopulle et al., 2021), changes in the number of cargos transported at a given time within a cortical neuron is expected to dramatically impact mRNA translation platform either in dendrites or in axons. In particular, the regulation of protein synthesis and degradation at the neuronal synapse is local and dynamic and modify the synaptic proteome autonomously during plasticity (Giandomenico et al., 2021). Hence, the synaptic function can be impacted if the number of moving lysosomes is affected.”

Point 10: In the Discussion, the section on “Potential druggable targets” is misleading. There is no clear delineation about which enzyme or pathway would be the target for such therapies nor how they would be leveraged to develop treatments for disorders associated with protein aggregation.

Reply: We replaced the Discussion section title “Potential druggable targets” by “Potential application in drug discovery assay” and rewrote the first paragraphs, while adding the new reference (Park et al., 2021) [page 22-23, lines 557-565]:

"We report here the use of a model based on primary mouse cortical neurons where it is possible to identify a robust decrease in the number of vesicles moving intracellularly (Figs. 2B, 3B, 4B and 5B), in the order of 30-50%, and up to 5-fold for lysosomes in neurons exposed to AßO (Fig. 5B). Such a large decrease is likely to impact the physiology of neurons and it is reasonable to consider that the transport of other cargoes, such as mitochondria and RNA granules, is also affected. This endolysosomal transport impairment endophenotype can be instrumental in generating large-scale drug-discovery campaigns (i.e., >105 compounds) as used in more complex human cellular models (Park et al., 2021).”

References added

Giandomenico SL, Alvarez-Castelao B, Schuman EM (2021) Proteostatic regulation in neuronal compartments. Trends Neurosci 3:S0166-2236(21)00161-2.

Park J-C, Jang S-Y, Lee D, Lee J, Kang U, Chang H, Kim HJ, Han S-H, Seo J, Choi M, Lee DY, Byun MS, Yi D, Cho K-H, Mook-Jung I (2021) A logical network-based drug-screening platform for Alzheimer’s disease representing pathological features of human brain organoids. Nat Commun 12:280.

Sardana R, Emr SD (2021) Membrane Protein Quality Control Mechanisms in the Endo-Lysosome System. Trends Cell Biol 31:269-283.

Schindelin J, Arganda-Carreras I, Frise E, Kaynig V, Longair M, Pietzsch T, Preibisch S, Rueden C, Saalfeld S, Schmid B, Tinevez J-Y, White DJ, Hartenstein V, Eliceiri K, Tomancak P, Cardona A (2012) Fiji: an open-source platform for biological-image analysis. Nat Methods 9:676-682.

Winckler B, Faundez V, Maday S, Cai Q, Almeida CG, Zhang H (2018) The Endolysosomal System and Proteostasis: From Development to Degeneration. J Neurosci 38:9364-9374.

Other changes

We reorganized the main text according to eNeuro style and placed the “Materials and Methods” section before the “Results”.

We also added a “Significance Statement” (page 3).
